# Somatic mosaicism in the diseased brain

**DOI:** 10.1186/s13039-022-00624-y

**Published:** 2022-10-21

**Authors:** Ivan Y. Iourov, Svetlana G. Vorsanova, Oxana S. Kurinnaia, Sergei I. Kutsev, Yuri B. Yurov

**Affiliations:** 1 Yurov’s Laboratory of Molecular Genetics and Cytogenomics of the Brain, Mental Health Research Center, Moscow, Russia; 2 Vorsanova’s Laboratory of Molecular Cytogenetics of Neuropsychiatric Diseases, Veltischev Research and Clinical Institute for Pediatrics and Pediatric Surgery of the Pirogov Russian National Research Medical University of the Russian Ministry of Health, Moscow, Russia; 3 Department of Medical Biological Disciplines, Belgorod State University, Belgorod, Russia; 4 Research Centre for Medical Genetics, Moscow, Russia

**Keywords:** Brain, Somatic mosaicism, Aneuploidy, Copy number variations, Gene mutations, Genome instability, Chromosome instability

## Abstract

It is hard to believe that all the cells of a human brain share identical genomes. Indeed, single cell genetic studies have demonstrated intercellular genomic variability in the normal and diseased brain. Moreover, there is a growing amount of evidence on the contribution of somatic mosaicism (the presence of genetically different cell populations in the same individual/tissue) to the etiology of brain diseases. However, brain-specific genomic variations are generally overlooked during the research of genetic defects associated with a brain disease. Accordingly, a review of brain-specific somatic mosaicism in disease context seems to be required. Here, we overview gene mutations, copy number variations and chromosome abnormalities (aneuploidy, deletions, duplications and supernumerary rearranged chromosomes) detected in the neural/neuronal cells of the diseased brain. Additionally, chromosome instability in non-cancerous brain diseases is addressed. Finally, theoretical analysis of possible mechanisms for neurodevelopmental and neurodegenerative disorders indicates that a genetic background for formation of somatic (chromosomal) mosaicism in the brain is likely to exist. In total, somatic mosaicism affecting the central nervous system seems to be a mechanism of brain diseases.

## Introduction

The human brain is a highly complex system encompassing ~ 100 billion neurons, up to 10^12^ glial cells and 5,000-200,000 synapses per neuron. Taking into account these astronomical amounts, neuronal and glial cells are unlikely to possess identical genomes [1, 2]. Accordingly, it has been proposed that somatic mosaicism (the presence of genetically (genomically) different cell populations in the same individual or tissue) in the brain may be a mechanism for neuronal variability in health and disease [3–7]. Somatic mosaicism encompasses all types of intercellular genetic variations. Somatic chromosomal mosaicism (the presence of cell population differing with respect to their chromosome complements) is one of the commonest types of somatic mosaicism. Single gene sequence variations (mutations), copy number variations (CNVs) and retrotransposition of transposable elements also represent common types of genetic variation involved in somatic mosaicism [8–10]. Finally, chromosomal instability (increased rates of non-specific chromosome abnormalities in a cell population) may be an underlying mechanism for the development of the genomically mosaic brain [6, 9, 11–13]. Generally, it is proposed that chromosomal instability and increased rates of somatic mosaicism (i.e. higher than in control brain samples or > 1–12% of genomically abnormal brain cells) are likely to cause brain disorders [14–18].

During the last two decades, a large amount of data generated by genetic studies of brain cells has been reported. Consequently, somatic mosaicism has been associated with neurodegenerative diseases (e.g. Alzheimer’s disease), autism, epilepsy, schizophrenia, and monogenic intellectual disability [3, 5, 9, 11, 12, 15–17, 19–29]. Here, we overview data on somatic mosaicism (from single nucleotide variants to aneuploidy, or losses/gains of whole chromosomes) in the diseased brain.

## Somatic mosaicism in brain diseases

The development of molecular cytogenetic techniques for studying human interphase chromosomes at any stage of the cell cycle and at molecular resolutions has provided an opportunity for uncovering somatic chromosomal mosaicism in the normal and diseased brain [30–33]. In parallel, single cell analysis using whole-genome sequencing has allowed the assessment of genomic variability in the normal and diseased brain [34–39]. Using similar advanced technologies for whole-genome analysis, CNVs confined to the diseased brain have been found [36–38]. **Schizophrenia**: individual cases of schizophrenia have been shown to be associated with aneuploidy and structural chromosomal imbalances in the diseased brain [3, 40–43]. **Neurodegenerative diseases**: Alzheimer’s disease cases have been associated with brain-specific aneuploidy [12, 26, 28, 44–47]. Furthermore, chromosome instability manifested as aneuploidy or structural chromosomal abnormalities has been found to mediate neurodegeneration in several devastative brain diseases [12, 28, 45–51]. Somatic mosaicism for single gene mutations (i.e. single nucleotide variants with a proven pathogenic effect) has been associated with Alzheimer’s disease [52, 54].

Single gene mutations were also found in individual cases of **autism** [13] and **epilepsy** [24, 27]. Mosaic genetic changes of chromosome X affecting the brain have been hypothesized to produce preponderance of males among individuals with neurodevelopmental diseases (e.g. autism) [54, 55]. Somatic mosaicim generated by LINE-1 retrotransposition [9] has been shown to be involved in the pathogenesis of **schizophrenia** and monogenic **intellectual disability** [56].

Dynamic changes of mosaicism rates (i.e. ontogenetic changes of proportions between normal and abnormal cells) affecting the human brain have been also suggested to modulate human behavior [57–59]. Thus, chromosome instability (chromosome condensation defects or alterations to chromosome structure/morphology without microscopically visible changes of chromosomal DNA) has been shown involved in gulf war illness pathogenesis [60]. Additionally, changing of chromosomal/genomic mosaicism rates has been hypothesized to be involved in behavior variability (e.g. worsening or improvement of behavioral abnormalities; sporadic occurrence or cease of behavioral abnormalities) in health and neurobehavioral diseases [59]. Table 1 summarizes available data on somatic mosaicism and genome/chromosome instability manifesting as chromosome abnormalities, CNVs, LINE-1 retrotransposition and single gene mutations detected in the diseased brain [3, 15, 26, 35, 40–47, 52, 53, 61–94].

Summarizing data on somatic mosaicism in the diseased brain depicted by Table 1 allows to conclude: (1) somatic mosaicism seems to be an appreciable source for human brain morbidity; (2) spectrum of mosaicism types is truly wide (almost all types of genetic mosaicism are detectable in the diseased brain); (3) pathways affected by the mutated genes are disease-specific and might be intriguing drug targets.

**Table 1:** Spectrum of somatic mosaicism detected in neural cells of the diseased brain

Disease/Disorder	Type of genomic change	Brief description	Chromosome	Locus	Gene	Refs
Alzheimer’s disease	Single nucleotide variants	Low-level mosaic single nucleotide variants	1	1q42.13	*PS2*	[53]
			14	14q24.2	*PS1*	
			17	17q21.31	*MAPT*	
			21	21q21.3	*APP*	
	Single nucleotide variants	Brain-specific single nucleotide variants	2	2q32.2	*COL3A1*	[61]
			4	4q31.3	*LRBA*	
	Single gene mutations	Single gene autosomal dominant variants	11	11q24.1	*SORL1*	[53]
	Single nucleotide variants	Single nucleotide variants in the temporal cortex	1	1q32.2	*CD55*	[62]
	Single nucleotide variants	Pathogenic somatic mutation leading to a loss-of-function mutation	19	19p13.2	*PIN1*	[63]
	Single gene mutations	Accumulating of mosaic somatic mutations in autism/intellectual disability genes	20	20q13.13	*ADNP*	[64]
	Nucleotide repeat expansion	Hexanucleotide repeat expansions	9	9p21.2	*C9orf72*	[65]
	CNVs	CNVs affecting ~ 10% of cells	NS*	NS	NS	[52]
	CNV (gain)	Single gene amplification	21	21q21.3	*APP*	[66]
	CNV (gain)	Single gene gain	12	12q13.12	*PRPH*	[65]
	DNA content variation	Increased rates of DNA content variation (variations of DNA content in a cell suggested to hallmark aneuploidy/polyploidy)	—	—	*—*	[47, 67]
	Aneuploidy	Increased rates of aneuploidy	17	—	*—*	[45, 67]
	Aneuploidy (trisomy/monosomy)	Chromosome-specific (numerical) instability	21	Whole chromosome	*—*	[15]
	Aneuploidy (monosomy)	X chromosome loss (an aging marker)	X	Whole chromosome	*—*	[46]
	Aneuploidy (chromosome instability)	Chromosome missegregation and aneuploidy probably resulted from mutations in the *APP*, presenelin 1 and, probably, *NPC1*	21	—	*—*	reviewed by [26]
Amyotrophic lateral sclerosis (sporadic)	CNVs	Brain-specific CNVs	3	3p26.3p26.2	*CNTN4*	[68]
			8	8p23.2	*CSMD1*	
			22	22q11.22	*GGTLC2*	
Ataxia telangiectasia (*ATM* mutations)	LINE-1 retrotransposition	*S*pecific LINE-1 retrotransposition	—	—	—	[69]
	Aneuploidy (chromosome instability) and chromosome 14-specific instability (affecting exclusively this chromosome)	High rates of chromosome instability in degenerating areas of the brain suggested to have *ATM* mutations (aneuploidy, non-random chromosomal breaks, rearranged chromosomes)	1, 7, 11, 13, 14, 17, 18, 21, X, Y	Whole chromosomes	*—*	[15, 44]
			14	14q12	*NOVA1, FOXG1B*	
Autism spectrum disorder	Single gene mutations	Recurrent deleterious mutations	2	2q24.3	*SCN1A*	[35]
			2	2q24.3	*SCN2A*	
			3	3p21.31	*SETD2*	
			6	6q25.3	*ARID1B*	
	LINE-1 retrotransposition	LINE-1 overexpression in the cerebellum	—	—	*—*	[70]
Focal Cortical Dysplasia	Single nucleotide variants	Missense mutations	9	9q34.13	*TSC1*	[71, 72]
			16	16p13.3	*TSC2*	
			1	1p36.22	*MTOR*	[73]
			22	22q12.2q12.3	*DEPDC5*	[74]
Focal cortical dysplasia, type II	Single nucleotide variants	Somatic doublet mutation	7	7q36.1	*RHEB*	[75]
Hemimegalen-cephaly	Single nucleotide variants	Missense mutations	1	1q43q44	*AKT3*	[71, 76]
			3	3q26.32	*PIK3CA*	
			14	14q32.33	*AKT1*	
	Single nucleotide variants	Missense mutations	1	1p36.22	*MTOR*	[73]
	Single nucleotide variants	“Double-hit” single nucleotide variants of two genes	1	1p36.22	*MTOR*	[77]
			9	9p22.1	*RPS6*	
Hypothalamic Hamartoma	Single nucleotide variants	Missense mutations	7	7p14.1	*GLI3*	[78, 79]
			X	Xp22.2	*OFD1*	
Frontotemporal lobar degeneration	Aneuploidy (trisomy)	Neuronal aneuploidy + apoptosis due to mitotic defects caused by *MAPT* mutations	12, 21	—	*—*	[80]
Huntington’s disease	Nucleotide repeat expansion	Expansion of an unstable trinucleotide repeat (CAG)	4	4p16.3	*HTT*	[81]
Lewy body diseases	Aneuploidy (NS)	Increase in neuronal DNA content (probably aneuploidy)	—	—	*—*	[82]
Niemann-Pick disease, type C1 (*NPC1* mutations)	Aneuploidy (trisomy)	Accumulation of (trisomic) cells with additional chromosome 21 in Niemann-Pick disease, type C1	21	—	*—*	[83]
Nonlesional focal epilepsy	Single gene mutations	Missense mutations, deletions (frameshift), insertions	X	Xp11.23	*SLC35A2*	[84]
Parkinson’s disease	Single nucleotide variants	Questionable *SNCA* variants	4	4q22.1	*SNCA*	[85]
	CNV (gains)	Somatic *SNCA* gains in nigral dopaminergic neurons	4	4q22.1	*SNCA*	[86]
Rett syndrome (*MECP2* mutations)	LINE-1 retrotransposition	Specific LINE-1 retrotransposition	—	—	—	[87]
Schizophrenia	Single nucleotide variants	NS	NS	NS	NS	[88]
	CNV (loss)	Somatic deletions	2	2q31.2	*PRKRA*	[42]
			5	5q35.2	*BOD1*	
			7	7p15.2	*CBX3*	
	CNVs (gains/losses)	Diseases-specific CNVs	4	4q35.2	NS	[43]
			6	6p11.2		
			7	7q11q12		
			11	11p15.4p15.5		
			15	15q11.2		
	LINE-1 retrotransposition	Increased LINE-1 “burden” and LINE-1 insertions in synapse or schizophrenia-related genes	—	—	*—*	[89, 90]
	Aneuploidy (trisomy)	Low-level mosaic trisomy	18, X	—	*—*	[3]
	Aneuploidy (trisomy/monosomy)	Low-level mosaic trisomy and monosomy	1	Whole chromosome	*—*	[40]
	Aneuploidy (trisomy/monosomy)	Increased rates of gonosomal aneuploidy	X, Y	—	*—*	[41]
Sturge-Weber syndrome (leptomeningeal angiomatosis)	Single nucleotide variants	Missense mutation (R183Q)	9	9q21.2	*GNAQ*	[91]
Subcortical band heterotopia (“double cortex” syndrome)	Single gene mutations	Mosaic gene mutations associated with the syndrome	17	17p13.3	*PAFAH1B1* *(LIS1)*	[92, 93]
			X	Xq23	*DCX*	
Tuberous Sclerosis	Single nucleotide variants	Missense mutations	16	16p13.3	*TSC2*	[94]

* NS — non-specific;

## Consequences and origins of somatic mosaicism in the brain

It is important to note that somatic mosaicism is detectable in biopsies of healthy individuals [57, 95, 96]. Analogously, from 0.5 to 12% of genomically abnormal cells are consistently detected in the unaffected brain [15, 17, 19, 23, 30, 34, 97]. However, clinical cohorts (including cohorts of individuals with neuropsychiatric disorders) generally exhibit high rates of somatic mosaicism, which seems to be involved in the pathogenesis [23, 57, 95, 98–100]. Nonetheless, despite debates regarding mosaicism rates in the normal brain, it is generally accepted that these are likely to be higher in the diseased brain [4, 5, 15, 23, 101, 102]. Furthermore, genomically abnormal (aneuploid) neurons have been demonstrated to be functionally active and integrated into brain circuitry [103]. On the other hand, the Alzheimer’s disease brain has not demonstrated increased rates of somatic mosaicism in a case-control study [104]. Still, there are a number of studies demonstrating mosaic mutations (single nucleotide variants and CNVs) of genes mutated in familial Alzheimer’s disease in the diseased brain [52, 66]. Taking into account the complexity of the disease, one can suggest that genome/chromosome instability and somatic mosaicism may be a mechanism for a proportion of cases [23, 28, 48]. Additionally, somatic mosaicism and genome instability have been systematically integrated into molecular and cellular pathways of neurodegenerative and neuropsychiatric disorders [12, 26, 28, 29, 38, 105–109]. Bioinformatics analyses and functional genomics studies have indicated that numerous mosaic (brain-specific) gene mutations are pathogenic [24, 39, 63, 92, 107]. Since chromosomal instability, structural variations and aneuploidy significantly affect cellular homeostasis, it has been systematically proposed that somatic mosaicism in the brain is able to cause central nervous system dysfunction or progressive neuronal loss [4, 5, 16, 19, 26, 45, 110]. However, there is an urgent need for forthcoming studies dedicated to functional consequences of somatic mosaicism in the brain.

A large part of human brain cells are generated during prenatal development without systematic/general renewal of neuronal cell populations after birth. Therefore, it is not surprising that the genetic landscape of the human brain is determined during early ontogeny stages [1, 23]. The developing human brain exhibiting high rates of chromosome instability and mosaicism manifested as aneuploidy [14, 16, 97]. Since these genetic variations are able to underlie neuronal cell death (for details see [110] and [111]) and a progressive decrease of cell numbers is observed in the developing brain during early ontogeny stages [1], it has been suggested that developmental chromosome instability and/or mosaicism underlying programmed cell death might be a regulation mechanism of cell numbers in the mammalian brain [14, 112]. Thus, alterations to pathways regulating programmed neuronal cell death might be responsible for the presence of abnormal cells in the postnatal brain [4, 22]. It is noteworthy that genomically abnormal neuronal cells are prone to cell death [110]. More precisely, aneuploid neurons selectively die in the diseased brain [45] and are susceptible to caspase-mediated death (e.g. apoptotic cell death) [110]. DNA damage in neurons may initiate apoptosis or produce a senescence-like state mediated by chromosome instability [113, 114], which may lead to cell death by another mechanism (e.g. mitotic catastrophe) [115]. Programmed neuronal cell death is a likely mechanism for neurodegeneration in aging-related brain diseases [45, 111, 115]. Furthermore, the genomic variations in the aged brain appear to underlie brain aging and aging-related brain deterioration [12, 25, 115–117]. Additionally, aging-related pathogenic processes in the Alzheimer’s disease brain may be associated with X chromosome aneuploidy, a chromosomal hallmark of human aging [46, 117,118]. A devastative consequence of alterations to programmed neuronal cell death may be the persistence of chromosome instability during early postnatal period, which is able to cause cancer in addition to non-cancerous brain diseases [119, 120]. Alternatively, the persistence of cell populations with altered genome is able to cause non-cancerous brain diseases [4, 5, 16, 37]. Thus, brain-specific somatic mosaicism is likely to result from developmental genomic instability and its rate fluctuations throughout ontogeny in an appreciable proportion of brain disease cases.

**Fig. 1:**
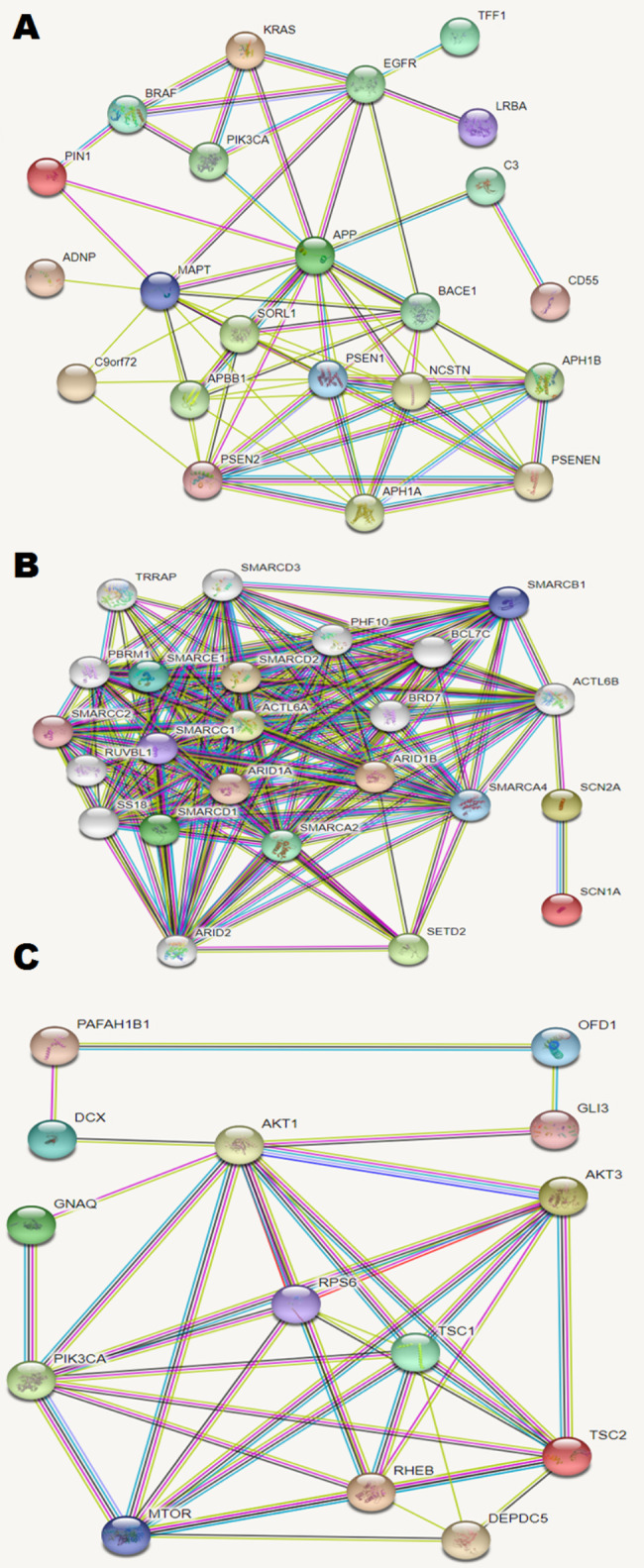
Interactomes of genes involved in somatic genome variations affecting the diseased brain (see Table 1) generated by STRING v11 [151]: (A) Alzheimer’s disease; (B) autism; (C) epileptic disorders

Alzheimer’s disease has long been associated with aberrant cell cycle (i.e. cell cycle re-entry, deregulation or endoreduplication) of neuronal cells [45, 47, 48, 121–124]. Mutations in the *APP* found in familial cases of Alzheimer’s disease may also cause chromosome mis-segregation leading to aneuploidy [121]. Similarly, *MAPT* mutations associated with frontotemporal lobar degeneration have been shown to produce mitotic defects in neuronal cells resulting in chromosome instability or aneuploidy [80]. Cohesion defects have also been associated with chromosome instability/aneuploidy in the Alzheimer’s disease brain [123]. Moreover, DNA replication stress [122] and genomic changes (CNVs) of genes implicated in the cell cycle pathway [125] are likely to be involved in molecular and cellular pathways to brain-specific somatic mosaicism (i.e. pathways of cell cycle regulation and mitotic checkpoint). It appears that these abnormal molecular and cellular processes leading to genome instability are similar to those observed in cancers [126, 127]. Oncogenic parallels are repeatedly noted in neurodegenerative diseases [28, 124]. However, “neurodegenerative” genomic instability originates from interactions between altered genome (mutational burden) and environment rather than from clonal evolution in cancers [28]. A recent study has shed light on a new formation mechanism of mosaicism for structural variations involving the *APP* gene in the Alzheimer’s disease brain, i.e. somatic gene recombination in neurons [128]. Finally, a more likely pathway to somatic mosaicism and genome instability in the brain includes specific genomic/genetic burden and the genetic-environmental interactions [129, 130]. In total, it seems that genes mutated in familial cases of complex brain disorders are involved in pathways of cell cycle regulation, mitotic checkpoint, chromatin remodeling, signaling (important for cell metabolism, proliferation and survival). To test briefly possible relevance of these assumptions, one may take a look at interactomes of genes listed in Table 1. Figure 1 demonstrates interactomes of genes involved in brain-specific somatic mosaicism in Alzheimer’s disease, autism and epileptic disorders. These diseases have been selected inasmuch as several genes have been repeatedly found mutated in the diseased brain. As one can see, these genes share same interactomic networks (apart from *COL3A1* and *PRPH* in Alzheimer’s disease). Moreover, elements of these interactomic networks are involved in a number of pathways, alterations to which might cause cellular genomes to become susceptible to the instability and to be dysregulated at the chromatin level. However, this is not the case for genes mutated in the schizophrenia brain (interactome cannot be generated). Unfortunately, studies aimed at analysis of genetic variation affecting genes of pathways implicated in maintaining genome stability, cell cycle regulation and programmed cell death are rare. Forthcoming studies of somatic genome variation in the brain are likely to be pathway-specific for unraveling the intrinsic causes of brain-specific mosaicism for diagnostic and therapeutic purposes [131].

## What is now and what is next?

Recent studies have additionally supported the idea that somatic mosaicism in the diseased brain may be a mechanism for neurodevelopmental and neurodegeneration disorders [132–134]. Furthermore, clinical (neurodevelopmental) cohorts repeatedly demonstrate high rates of somatic mosaicism [135–137], which may be seen as a mechanism for the disease or a target for therapeutic interventions [23, 138]. In this context, it is to mention an intriguing mechanism for brain-specific chromosome instability and/or aneuploidy termed chromohelkosis (chromosome ulceration or open wound), which results from the co-occurrence of non-mosaic and mosaic chromosome imbalances caused by a susceptibility to genome instability and a genomic rearrangement [139]. Therefore, as noticed previously [129], environmental interactions with changed genomes should not be left aside in studies dedicated to somatic cell genomics of brain disorders. To support this idea, one can refer to the ability of the notorious COVID-19 virus to produce aging-related genome/chromosome instability in the diseased brain [140]. Thus, therapeutic interventions based on analysis of brain-specific somatic mosaicism are to be developed taking into account genetic-environmental interactions.

Successful therapeutic interventions in brain disorders mediated by somatic mosaicism appear to require specific diagnostic approaches. Emerging technologies based on genome scanning techniques, molecular cytogentic/cytogenomic methods and post-genomic bioinformatic approaches are likely to be the way for the success [131, 141–143]. Cytopostgenomics and systems cytogenomics seem to be the areas of cytogenetic research which would help to develop the approaches to uncover causes and consequences of somatic chromosomal mosaicism in the diseased brain [142, 143]. Analyzing available candidate processes or pathways for therapeutic interventions in brain disorders mediated by somatic mosaicism (e.g. DNA reparation, programmed cell death, neurodegeneration pathway) [144–146] gives an opportunity to suggest that pathway-centric (cyto)genomic studies are likely to be the most promising. To this end, it is to note that modern molecular cytogenetic and genomic techniques are able to generate new data on the role of somatic mosaicism in the aging and diseased brain [147–150].

## Concluding remarks

Technological advances in sequencing resulted in an overuse of molecular (sequencing) methods comparing to molecular cytogenetic techniques. As a result, little attention is paid to molecular cytogenetic aspects of somatic mosiacism in the human brain. This review represents a unique overview of both molecular genetic and molecular cytogenetic (cytogenomic) data on brain-specific genomic variations at DNA, subchromosomal and chromosomal levels associated with a wide spectrum of non-cancerous brain diseases.

Regardless of a relatively small amount, studies dedicated to somatic mosaicism in the human brain have demonstrated a wide spectrum of genomic variations involved in neurological and psychiatric diseases. Brain-specific genome variations causing neurodegenerative and neuropsychiatric disorders produce several important tasks for current biomedicine. Firstly, the unavailability of tissues for premortem genomic analysis (apart from surgical biopsies) raises important diagnostic issues. Here, we have suggested that a kind of susceptibility of cellular genomes to become unstable (i.e. mutations of genes involved in molecular and cellular pathways to maintain genomic stability throughout cell cycle) appears to exist. Briefly, (i) in the developing human brain, chromosome instability and mosaic aneuploidy/CNVs have a high rate, which is, however, significantly diminished in the postnatal brain; (ii) alterations to pathways of genome stability maintenance, cell cycle regulation and programmed cell death should mediate the persistence or increase of genome instability (mosaicism) rates in the brain; (iii) this persistence/increase affecting a proportion of brain cells may cause central nervous system dysfunction or neuronal loss (brain diseases).

Uncovering the susceptibility to brain-specific chromosome/genome instability might have diagnostic value. Moreover, these pathways to brain-specific genome instability and somatic mosaicism may be a drug target in brain diseases mediated by somatic mosaicism. Actually, pathogenic cascades of brain diseases involving somatic mosaicism and genome instability are suggested to be valuable drug targets. Furthermore, data on somatic mosaicism in surgical biopsies have already been considered useful for the therapeutic interventions. The exciting area of somatic cell genomics brings new insights into genetic (genomic) mechanisms of brain dysfunction, which are required for efficient molecular diagnosis and treatment of neurological and psychiatric illnesses.

## Data Availability

Not applicable.
